# Rheology Applied to Microgels: Brief (Revision of the) State of the Art

**DOI:** 10.3390/polym14071279

**Published:** 2022-03-22

**Authors:** Coro Echeverría, Carmen Mijangos

**Affiliations:** Instituto de Ciencia y Tecnología de Polímeros (ICTP-CSIC), C/Juan de la Cierva 3, 28006 Madrid, Spain; cmijangos@ictp.csic.es

**Keywords:** microgels, rheology, scaling theories, applications

## Abstract

The ability of polymer microgels to rapidly respond to external stimuli is of great interest in sensors, lubricants, and biomedical applications, among others. In most of their uses, microgels are subjected to shear, deformation, and compression forces or a combination of them, leading to variations in their rheological properties. This review article mainly refers to the rheology of microgels, from the hard sphere versus soft particles’ model. It clearly describes the scaling theories and fractal structure formation, in particular, the Shih et al. and Wu and Morbidelli models as a tool to determine the interactions among microgel particles and, thus, the viscoelastic properties. Additionally, the most recent advances on the characterization of microgels’ single-particle interactions are also described. The review starts with the definition of microgels, and a brief introduction addresses the preparation and applications of microgels and hybrid microgels.

## 1. Introduction

**Figure 1:**
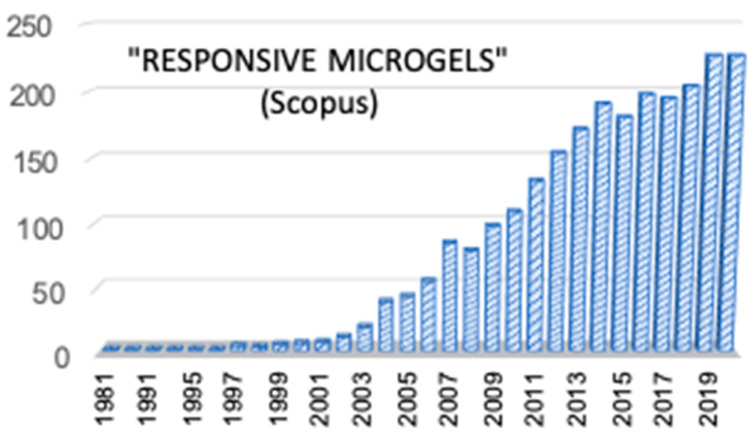
The evolution of the citations about responsive microgels up to 2020 are shown (source: Scopus).

In 1949 Baker et al. coined for the first time the word *microgels* referring to cross-linked polybutadiene latex particles [1]. However, microgels already existed before, since the beginning of the industrial polymer synthesis in the 1920s, and were recognized as undesired subproducts derived from the synthesis of elastomers. Microgels are defined as intra-molecularly cross-linked polymer particles in a colloidal size range. This definition is widely accepted and brings implicitly the following: (1) the small particle size range of 10–1000 nm, which is typical of colloidal particles, and (2) they are dispersed in a solvent [2,3,4,5,6]. Additionally, microgels are in an intermediate step between branched polymers and macroscopic networks, which implies that in a suitable solvent they swell instead of dissolving, thus forming a colloidal dispersion, as mentioned. They are also considered as soft particles with the ability to rapidly respond to variations in their environment, resulting in changes in their polymer chain conformation. Furthermore, depending on the building monomer, on the composition, and on the added chemical functionalities, microgels can be sensitive to external stimuli such as temperature, pH, and ionic strength, among others, thus revealing their versatility and potential applications further described in this review. In order to highlight the relevance of microgels within polymer science, in Figure 1 the evolution of the citations about responsive microgels up to 2020 is shown.

### The Preparation of Microgels

The key to the diverse microgel systems’ versatility, functionality, and further applications is founded in the preparation step. In this regard there are already several recent reviews published covering different aspects of microgel preparation, design, and synthesis [7,8,9]. Our intention with this review was to give the reader a glance at the most widely used polymerization techniques and actual methods to obtain tailored microgels using a few examples based on our work and work done by relevant research groups in the field.

The most widely used methods of preparation based on free-radical emulsion methods are precipitation polymerization and inverse emulsion (water in oil (W/O), water in oil in water (W/O/W)). These methods provide versatility and, what is more important, the possibility to scale up the production [7]. The precipitation polymerization method, also known as surfactant-free emulsion polymerization, is generally used for the obtainment of hydrophilic microgels during in situ cross-linking of the precursors in reservoir water droplets free of surfactant. The first research work done following this preparation strategy was reported by Pelton and Chibante and was used for the synthesis of poly(N-isopropylacrylamide) (PNIPAM) microgels by means of precipitation polymerization [10]. This early work paved the way for the preparation of innumerable microgel systems based on precipitation polymerization. Indeed, it has also been applied for the development of acrylamide-based monomers, such as the already mentioned representative N-isopropylacrylamide (NIPAM) that has been undoubtedly the most studied one for the development of thermoresponsive microgels. Much of the research based on PNIPAM microgels has been done by Richtering et al. [11,12,13,14,15], Vincent et al. [3,16,17], and Snowden et al. [18,19,20] among others, as reflected in the great number of publications that deal with their preparation and characterization. Similarly, Pich et al. [21,22,23] and Forcada et al. [24,25,26,27,28,29] among other groups have also used the precipitation polymerization method for the development of N-vinylactam monomer-based microgels such as poly (N-vinylcaprolactam) (VCL); as with PNIPAM, these are also known for their thermoresponsive behavior in aqueous solution. Regarding the acrylamide-based monomers, Mijangos et al. took advantage of the inverse emulsion polymerization method (W/O) to prepare polyacrylamide (PAAm)-based microgels that also resulted to be thermoresponsive, showing an upper critical solution temperature (UCST)-like behavior; microgels collapse upon heating. It is worth mentioning that the inverse emulsion polymerization method allows the encapsulation of active compounds and/or inorganic nanoparticles as one of the strategies for the development of hybrid microgels. This strategy opens the way toward multifunctionality and multi-responsiveness in microgels, as will be further mentioned. One of the advantages of using emulsion polymerization methods is the possibility to introduce additional functional groups through the incorporation of comonomers to the reaction [30]. The most typical comonomers that have been incorporated into microgel synthesis are the organic acids (methacrylic acid (MMA), acrylic acid (AA), and dimethylaminoethylmethacrylate (DMAEMA), which confer pH responsiveness.

Beyond the classical emulsion polymerization method, many researchers in the last few years have turned their strategies toward living radical polymerization techniques such as, for instance, the reversible addition-fragmentation chain transfer (RAFT) method. An example of this can be found in the research done by Pitch et al., where PNIPAM-based microgels were prepared by RAFT-mediated precipitation polymerization using a zwitterionic sulfobetaine macro-RAFT. This controlled polymerization method was derived in highly monodisperse microgels decorated with polyzwitterionic chains [31]. More recently, Etchenausia et al. synthesized a reactive cationic polymer (poly([2-(acryloyloxy)ethyl]trimethylammonium chloride) as a macro-RAFT agent for the subsequent precipitation polymerization of poly(N-vinylcaprolactam) (PVCL) microgels [32]. The improvement produced by the macro-RAFT agent was evidenced when comparing the colloidal features of the PVCL microgels with those synthesized by using the nonreactive cationic polymeric or molecular stabilizers.

The evolution arisen from the control over the microgel synthesis (monomer and cross-linker reactivities) allowed the design of different microgel architecture: core-shell, multicore-shell, and hollow microgels, among others [33,34,35,36,37]. In that way, microgels with improved properties and additional functions can be prepared; these could respond, i.e., swell and/or shrink, in a controlled and desired manner to environmental changes or be programmed to have a particular swelling behavior. An example of this can be found in the work of Brugnoni et al., with the production of core-shell-shell thermoresponsive microgels with silica nanoparticles in the core, PNIPAM in the outer shell, and poly(N-isopropylmethacrylamide) (PNIPMAA) as the inner shell [38]. Taking advantage of computer simulation, the core-shell-shell microgel system revealed unique behavior: the inter-penetration among the two outer layers. This significant interaction is associated with different responses to temperature of each layer. This means that the swelling capacity can be controlled by using temperatures as external stimuli so that different and desired swelling states could be achieved. Another example of the relevance of that architecture can be found in the work of Saha et al., in which a dual stimuli-responsive antifouling microgel system was designed with tunable UCST-LCST transition temperatures [39]. The obtained microgels have the potential to be used as coatings or additives in biomaterial devices.

## 2. Applications of Microgels

**Figure 2:**
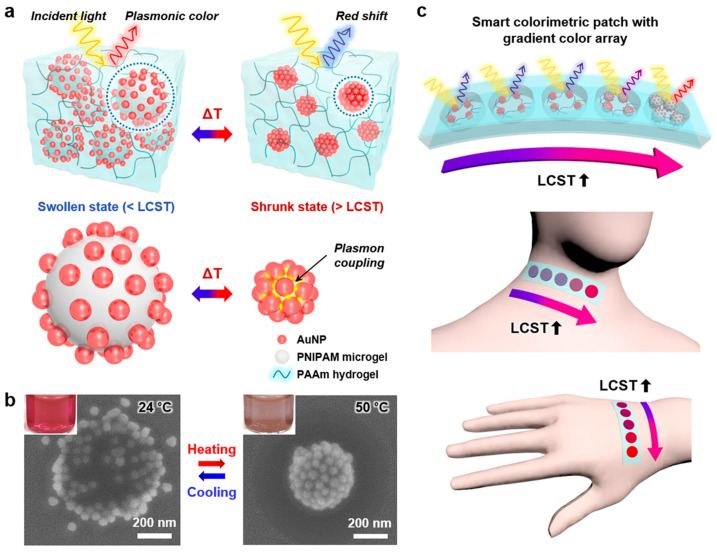
(**a**) Schematic illustration of the plasmonic microgels in the PAAm hydrogel under swollen and shrunken states. (**b**) SEM images of the plasmonic microgels with 51-nm AuNPs under the swollen state at 24 °C (left) and under the shrunken state at 50 °C (right); the inset images show pictures of the plasmonic microgel dispersions under each condition. (**c**) Schemes of the sensor array patches attached to human skin at different positions (neck and hand). (Reprinted from Ayoung Choe et al. [47]; licensed under Creative Commons CC BY li (open access)).

Following this trend, researchers in the field have dedicated much of their effort to obtain advanced materials from stimuli-responsive polymeric microgels, thus widening the range of potential applications. For this purpose, one of the strategies followed in the last decades consisted of the design and preparation of hybrid microgels. In this sense, the first work of Antonietti et al. [40], where microgels acted as a microreactor for the controlled growth of gold nanoparticles, could be considered as the tipping point from which the research on the development of hybrid microgels grew significantly. It is also worth highlighting the pioneering work done by the Ballauff group, which strongly contributed to developing novel nanoparticle/microgel systems and to gaining knowledge on this topic [41,42,43,44,45]. The incorporation of inorganic nanoparticles into stimuli-responsive microgels, such as gold or magnetic nanoparticles, provides additional functionalities, such as light or magnetic responsiveness, respectively [46]. For instance, in a recent work Choe et al. developed a smart colorimetric thermoresponsive patch based on thermoresponsive plasmonic microgels embedded within a hydrogel matrix. The objective of the work was to achieve a fast and efficient thermoresponsive color change for which raspberry-shaped plasmonic microgels were fabricated by decorating gold nanoparticles (AuNPs) on poly(N-isopropylacrylamide) (PNIPAM) microgels. Microgels exhibited reversible color shifts in response to temperature changes and to stretchability [47]. These colorimetric patches, with tunable critical temperatures, enable sequential responses by means of color changes and movements in response to temperature, which opened up the possibility to use them in applications as smart sensors, actuators, and soft robotics. As a proof-of-concept, the authors developed a smart hand that exhibited sequential responses to color changes and movements of fingers in response to environmental temperature changes (Figure 2) [47].

Another example can be found in the recent work of Serpe et al. with the synthesis of dual stimuli-responsible hybrid PNIPAM microgels with AuNPs in the core. These AuNPs’ cores served to stimulate the collapse of the PNIPAM shell when excited with visible light. This visible light generates heat that causes the swelling of PNIPAM [48]. Regarding hybrid microgels containing magnetic nanoparticles, Rames et al. developed a platelet-like stimuli-responsive microgel suspension based on poly(NIPAM-co-AA) that responds to the magnetic field and UV photocuring. The objective of the work was to repair, in a remotely controlled way, nonwoven fiber mats composed of commercially available polypropylene (PP) and polybutylene terephthalate (PBT) in order to recover their native morphological and functional properties [34].

Throughout this introduction, besides the great versatility of polymeric microgels, it was evidenced that microgels have significantly contributed to the development of advanced materials for biomedical applications [49]. In the following paragraphs we will highlight the role of microgels in three different applications. Probably one of the most studied potential applications is their use as drug carriers and drug delivery systems. However, polymeric microgels have also emerged as building blocks that can self-assemble, rearrange, and interact to generate coatings, structures, and scaffolds that could serve as applications as relevant as tissue regeneration and biolubricants, among others.

### 2.1. Microgels in Drug Delivery Applications

In the field of the controlled release of active compounds, the role of nanotechnology has driven the development of micro/nanoscopic polymeric systems (polymer–drug conjugates, micelles, dendritic structures) among which microgels have a highlighted role. The possibility to tune the microgel mesh size, that is, porosity, in addition to the composition, colloidal stability, and elasticity, has significantly contributed to the development of microgels as drug delivery or active compound carriers [50,51,52,53]. These systems are the basis of what is now known as nanomedicine, whose research in the last decade has been mainly directed toward the development of theragnostic systems, a combination of a therapeutic and diagnostic functions. In this regard, hybrid microgels have been widely studied due to their multi-responsiveness, as it was recently described in a review article by Fernández-Barbero et al. [54]. As an example of hybrid microgels for theragnostic purposes, Jaiswal et al. [55] developed thermoresponsive magnetic hydrogels based on PNIPAM, which demonstrate multimodal imaging and the remote radio frequency-triggered release of therapeutic cargo (doxorubicin), which resulted in cell death. Similarly, in recent research, Vijayan et al. developed a magneto-fluorescent nanogel with a promising future potential for cancer theragnostic applications. These nanogels based on polyethyleneglycol (PEG) and DMAEMA microgels and superparamagnetic iron oxide nanoparticles (SPION), with a core-shell morphology (core for the SPIN and shell for PEG), showed magnetic hyperthermia (as a therapeutic component) and bioimaging properties (for diagnosis) [56]. There is no doubt that the evolution of nanomedicine is directed towards personalized medicine and towards the search for polymeric systems capable of synchronizing with the physiological state of the patient and responding to it. In short, research is being carried out on the development of molecular imprint polymers (MIPs), which are also known as synthetic antibodies. MIPs are polymeric matrices with specific cavities for molecular recognition. An example of these systems can be found in the work done by Seyfoori et al. in which hybrid immune microgels are developed as a tool for the magnetic isolation of targeted tumor cells [57]. These circulating tumor cells are of extreme relevance for biopsies as an indicator of metastasis. In order to do this, magnetic nanoparticles were synthesized in situ within the microgel thermoresponsive poly(N-isopropylacrylamide)-co-acrylic acid (PNIPAM−AA) matrix.

### 2.2. Microgels in Tissue Regeneration

In general, polymeric materials play a relevant role in the development of scaffolds, which is one of the three pillars of tissue engineering: scaffolds, cells, and environmental conditions. Scaffolds are designed to mimic a tissue’s extracellular matrix, for which a 3D structure is necessary as a structural support, but also in order to guide cell growth and final tissue regeneration. Certainly, microgels are already 3D systems that could contribute to guiding cell growth and fulfill the requirements indicated to overcome tissue regeneration [58]. Within this context microgels are currently being studied for various tissue engineering applications: cartilage, vascular, cardiac, hepatic, and neuronal tissues, among others [59], as in the aforementioned work of Serpe et al. in which they designed light-responsive microgels with potential bone tissue regeneration. These microgels are capable of loading and releasing dexamethasone (DEX), an active compound able to induce human mesenchymal stem cells’ (hMSCs) differentiation into osteoblast [48]. Another example of the use of microgels for tissue regeneration is the work of Rieder et al. on the development of injectable microgels densely packed into a microporous scaffold. These chitosan-based microgels are able to encapsulate two different growth factors that contribute to tissue regeneration. Additionally, interactions between microgels and endothelial cells were also studied: Endothelial cells were able to attach directly to the microgels and proliferate within the porous network created by the packed microgels [60]. Microgels can also be designed and tuned as cell carriers containing one or more cells, functioning as individual cell culture units. However, what is more interesting is that different fabrication strategies might enable hierarchical assembly of multiple kinds of microgels that allow building multifunctional scaffolds mimicking the heterogeneous extracellular environment that exists in vivo [61]. Within the field of advanced tissue engineering, a technique that is currently gaining interest is the additive manufacturing for 3D printing with living cells, extracellular matrix, and hydrogels; however, the disadvantage of these materials is the lack of physical support of the printed structures. Such structures suffered from a post-processing step (toughened chemically or mechanically). A recent alternative to this strategy is the use of jamming microgels able to keep the structure by trapping material in a space as precise as 1–2 cell diameter dimension [62,63]. Since one of the key aspects in 3D bioprinting is the rheology, in a recent research O’Bryan et al. were able to tune the rheological behavior of jamming microgels in order to accomplish 3D printing of different structures and potential scaffolds with high precision [64].

### 2.3. Microgels as Biolubricants

**Figure 3:**
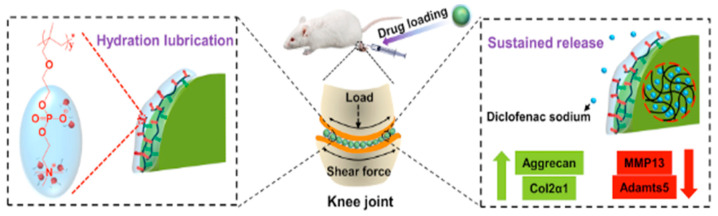
Schematic representation of the intra-articular injection of drug-loaded lubricant microgels for the treatment of osteoarthritis. Adapted from Han et al. [69]; licensed under CC BY 4.0 (open access).

In the last decades, colloidal microgels have gained notoriety in the development of biological lubricants. Biolubricants’ main function is to hamper the joints from suffering friction during movements. When this biolubrication fails it can cause important damage in the cartilage and, in the worst scenario, this could end up in a replacement of the joint. The lubricity is mainly originated from the contribution of two different constituents, which are cartilage and synovial fluid [65]. From the point of view of material science, the cartilage can be considered as a hydrogel due to its elasticity and softness. In this regard, researchers have performed a large number of studies on hydrogel tribology in seeking materials with an ultra-low coefficient of friction and high load [66,67,68]. However, it is well established that mechanical properties of hydrogels offering good lubricant property are poor, thus limiting their practical applications. Considering this problem, microgels represented an advance in the field due to the exhibited advantages based on (1) their colloidal stability, which ensures smooth water lubrication, (2) their viscoelasticity, which provides better compression (friction) resistance, and (3) the possibility of loading drugs, providing an additional therapeutic function [49]. An example of the potential that microgel systems might have as biological lubricants can be found in the recent work of Han et al. with the development of injectable microgels that demonstrated improved lubrication and controlled drug release for the treatment of osteoarthritis [69] (see Figure 3). Before this, Liu et al. synthesized magnetite-loaded thermoresponsive PNIPAM-based microgels able to reduce friction when used as an additive in aqueous lubricants. The relevance of this research is centered on the ability to remotely control, by temperature, magnetic field, and light, the colloidal properties and, therefore, the friction occurring in the interface [70].

As mentioned in the different microgels’ potential applications herein described, their deformability and compressibility (viscoelastic properties) allow us to reach non-spherical shapes so that they can densely pack to form scaffolds or undergo inter-penetration only of their external shell (in a core-shell microgel architecture). Undoubtedly, microgels owe their properties mainly to their complex morphology (and architectures), inter- and intra-particle interactions and further packaging, which give rise to a wide variety of new physical states and behaviors [71]. Consequently, such advantages contribute to the production of advanced materials with different biomedical purposes, as in the examples shown above. This brief overview clearly evidenced that the performance of colloidal microgels in the different applications, particularly in scaffold formation and biolubrication, is directly dependent on their viscoelastic properties and on their rheological behavior; therefore, being able to fine-tune the interactions, inter-penetration, and self-assembly of microgels could contribute to improve the performance of the system in the required application. Accordingly, the intention of this review was to put the focus on the rheological aspect of colloidal microgels, focusing on the most recent research performed in this respect.

## 3. Rheology of Microgels

Microgel dispersions combine the properties of hydrogels with the particularities of colloidal systems; depending on their composition, they can respond to external stimuli (pH, temperature, light, magnetic field, etc.), which opens up their potential use for a wide range of applications. In most applications, microgels are subjected to conditions of shear, deformation, and compression, or their self-assembly is required. In all these situations, their behaviors under such conditions have a direct effect on the final material performance. Therefore, it is indeed crucial to understand and gain knowledge on the rheological properties of microgels’ dispersions.

### 3.1. Hard Spheres vs. Soft Particles

The rheological properties of hard particles are driven by the volume fraction of the dispersed phase within the suspension. Disordered hard sphere suspensions achieve their maximum volume fraction at a random close packing or jamming, ζ_rcp_ ≅ ζ_J_ ≅ 0.64. In contrast, suspensions of soft particles can deform, ζ_J_ ≤ ζ_J_ < 1 [72]. In this range, the particles form flat surfaces at interaction points, which successively store elastic energy, and result in soft pastes such as mayonnaise, shaving foams, and similar items [73,74]. However, microgels are different. As will be evidenced in this review, their physical nature lies between rigid spheres and ultra-soft colloids. Depending on their composition, swelling ability, and architecture, they can exhibit physical properties of both. In addition, as will be also described, microgels are compressible and deformable; this will enable another state in which microgel particles are overpacked [11,75,76].

Microgels are often studied by comparison to hard sphere model colloids due to the fact that these types of colloids were very well characterized [77,78,79]. As with hard spheres, microgel suspensions also undergo an entropically driven phase transition from liquid to crystal and they also form colloidal glasses [80,81,82]. Within this context, Graessly et al. studied the rheological properties of poly(acrylic acid) (PAA) microgel suspension and the effect of the microgel concentration on the rheological properties [83]. From this study it was evidenced that PAA microgels behave as elastic solids under deformation and have the ability to recover from such deformation below the yield point. They associate this result to the deformability of the microgels and the possibility of entanglement formation between microgels. Later, Wolfe and Scopazzi studied the effect of a cross-linking degree on the rheological properties of polymethylmetacrylate (PMMA) microgels [84]. The viscosity of the swollen microgels was found to scale with the effective volume fraction; but, it was also evidenced that for microgels with a low cross-linking degree the shear-dependent viscosity diverged from the behavior expected for hard spheres, revealing a softer character of the microgels. Following this result, Kiminta and Luckman took into account this softer character and considered microgels as a model for the rheology of soft and deformable particles frequently found in the industry [85]. In particular, the rheology of PNIPAM thermoresponsive microgels was studied in which a decrease in the viscosity with shear rate and shear thinning was observed. Additionally, even at low microgel concentrations, a drastic increase in the viscosity was observed, showing values almost three orders of magnitude higher than polystyrene latex [86]. This behavior is due to the swollen state of microgels having much higher effective volume fraction. A direct effect of microgel particle size on the viscosity of the dispersion was also detected. For the same concentration of microgels, smaller microgel particles resulted in lower viscosity values, which was in accordance with the behavior observed for hard spheres such as polystyrene latex [86].

Richtering’s group pioneered raising the question of whether microgels could be described from the point of view of hard spheres or if, on the contrary, they could be described from the optics of soft colloidal systems since microgels’ swelling property gives rise to soft and deformable particles, as mentioned [81]. To elucidate this question, they investigated the inter-particle interactions in concentrated suspensions of PNIPAM microgels of different sizes and cross-linking degrees and characterized the structure factors of these microgels assuming a volume fraction of a hard sphere. From this study it was determined that the microgel particles behave as hard spheres up to an effective volume fraction of 0.35. Above this value microgel particles experienced certain compression, being the reason they deviate from a hard sphere behavior and describing an elastic modulus plateau typical of soft spheres [87].

Therefore, and taking Richtering’s study into account, it seemed reasonable to think that in a static situation microgel particles in suspension could behave as hard sphere systems. However, when we imagine microgels in a dynamic situation, the plausible behavior diverges from that of hard spheres. A sphere with diffuse polymeric chains such as a microgel, under flow conditions, seems to behave differently than a hard sphere. When a colloidal suspension is sufficiently concentrated, the motion of an individual particle is no longer purely Fickian. Each particle interacts with the surrounding particles and its diffusion over a long distance slows down as the volume fraction increases. Generally, at high concentrations two characteristic times appear: At short times, the diffusion of the particles occurs in the inter-particle distance or within the “cage” formed by the contiguous particles. At long times, the diffusion is directly related to large distances. The slow mode exceeds the experimental time at a finite concentration, which leads to a dynamic arrest of the microgel particles resulting in the formation of a colloidal crystal. This effect has been theoretically described by the mode-coupling theory, among other models, which predicts a volume fraction glass transition below the experimental rigid sphere system [88]. Colloidal dispersions in such arrested situations behave differently under the effects of shear. At low strains, colloidal dispersions behave as an elastic solid but flow under stress above a critical stress or strain threshold [89]. This performance, which is typical of a large number of soft materials such as emulsions, foams, pastes, and foods, has attracted much interest due to such dual characters under deformation and also due to the fundamental physics behind this behavior [90].

### 3.2. Interactions among Microgel Particles

#### 3.2.1. Scaling Theories and Fractal Structure Formation in Colloidal Microgels

Since the 1980s, the dynamic arrest described in colloidal dispersions has been the focus of important study in the field of colloidal physics, in particular with the objective of understanding whether this arrested state also resulted in the formation of agglomerates or clusters derived from possible colloidal particle interactions [91,92]. This information is very relevant since it could directly affect the processing and performance of the material in the final application.

A colloidal system can be dispersed or flocculated depending on the energy of the interactions between particles and also the concentration [93,94]. The formation of clusters or agglomerates is a complex and random process that can result in self similarities or recurrence [95]. By means of computer simulation and subsequent experimental results, we can define the existence of two different growing mechanisms for the formation of clusters/agglomerates: diffusion-limited aggregation mechanisms (DLCA) and reaction-limited aggregation mechanism (RCLA) [96,97,98]. In the case of DLCA, the cluster aggregation is fast, deriving in a looser and ramified aggregated structure. In the case of RCLA, the aggregation regime is slow, resulting in aggregates with denser and compact structures. In addition, it has been theoretically determined that each growing mechanism is described by a specific fractal dimension value, approximately 1.7–1.8 and 2.0–2.2 for DLCA and RCLA, respectively; however, there is a large majority of systems that present intermediate aggregate formation mechanism [99,100,101,102].

**Scheme 1:**
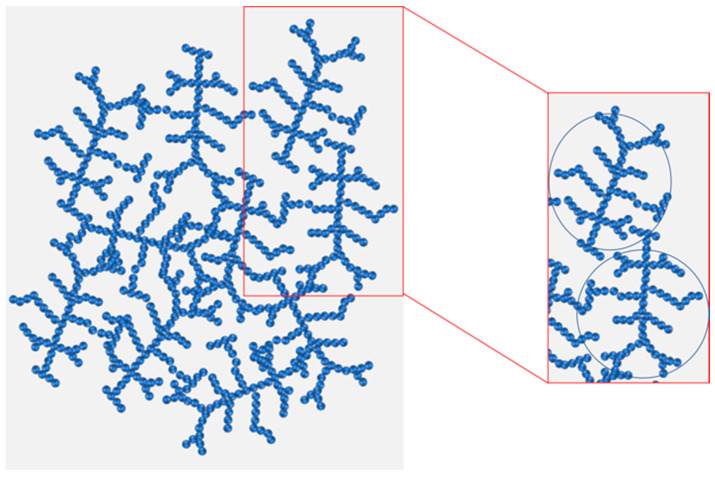
Schematic representation of an agglomerated microgel dispersion (**left**) and the formation of microgels’ fractal flocs or agglomerates (**right**).

Colloidal gels, three-dimensional (3D) network-like structures composed of aggregated colloidal particles in suspensions, can become a special state of a flocculated system. The structure of the colloidal gel is highly disordered; but, at certain length scales, they are often self similar and can be described in terms of fractal geometry (see Scheme 1). The formation of colloidal gels are common when using stimuli-responsive microgels since inter-particle interaction can change with external stimuli (temperature, deformation, pH) [103]. One way to analyze the structure of dispersed microgels would be through fractal analysis.

In order to determine the colloidal gel structure and correlate it to the rheological properties, Brown and Ball developed a scaling theory for the first time [104,105]. Later, this was extended by Shih et al., establishing a scale model based on concepts that were already applied for polymeric gels [106,107,108]. Ten years later, in 2001, Wu and Morbidelli [109] extended the Shih et al. model by introducing an additional parameter, α, the microscopic elasticity parameter, which allows us to obtain more information on the formation of fractal aggregates. Shih et al.’s and Wu and models are probably the most relevant scaling models. As so, they are explained below.

Shih et al. model [108].

The model considers that the fractal dimension of the aggregates, *D*, can be determined from the scale relationship between the mean size of the aggregates, *ξ*, and the particle concentration or volume fraction, *φ*; assuming the particle concentration within the aggregate *ξ* as the total concentration *φ*:

(1) $$\xi \sim \varphi^{1/\left(D-d\right)}$$

where *d* is the Euclidean or spatial dimension of the system.

The next step is to find the scale ratio of elastic constant *K_ξ_* of an *ξ* size aggregate. As mentioned, the elastic properties of the aggregates are determined by the structure of the set of particles that are attached to each other. In order to continue with the mathematical development, Shih et al. introduced an additional parameter defined by Kantor and Webman [107], which considered the structure, the set of particles, as a linear chain of *N* springs with an elastic constant *K_m_* defined as:

(2) $$K_{m}\sim \frac{K_{0}}{NS^{2}}$$

with K_0_ being the elastic constant of two contiguous springs, independent of the particle concentration, and *S* being the radius of gyration.

Translating this equation to colloidal gels, as the number of springs N a set of particles bound together with a size *ξ* were considered, with a radius of gyration comparable to that of the springs *S*; thus,

(3) $$N\sim \xi^{x}$$

with the exponent *x* being a number less than the fractal dimension of the aggregates, *D*, but greater than unity. Therefore, now *S*^2^ is the square of the size of the aggregates, *ξ*. Combining Equations (2) and (3), the elastic constant of the aggregates *K_ξ_* is obtained:

(4) $$K_{\xi }\sim \frac{K_{0}}{{\xi .}^{2+x}}$$

From Equation (4) it can be extracted that as the size of the aggregates increases the elastic constant of the aggregates decreases in an exponential manner with an exponent of 2 + *x*. This means that, as the aggregates grow, their behavior is more similar to that of weaker springs. Generalizing this behavior to an *L* size system with a *K* macroscopic elastic constant, this can be represented as a function of the individual elastic constant of the aggregates, as shown:

(5) $$K_{\;}\sim {\left(\frac{L}{\xi }\right)}^{d-2}K_{\;}$$

From Equation (5) it can be deduced that the elastic constant of the aggregates is dependent on both the size of the aggregates and the fractal dimension of the chain. Therefore, *K* is dependent on the strength of the interactions between the aggregates (inter-floc) and the strength between the particles themselves within the aggregates (intra-floc). The major conclusion drawn from the Shih et al. model is that it is possible to differentiate two extreme situations and unravel the intra- and inter-floc interactions in two separate regimes: a *strong-link regime,* where inter-floc interactions are stronger than intra-floc (among particles) interactions, and a *weak-link regime,* where interactions among particles inside the floc are stronger and determine the elasticity of the system.

**Scheme 2:**
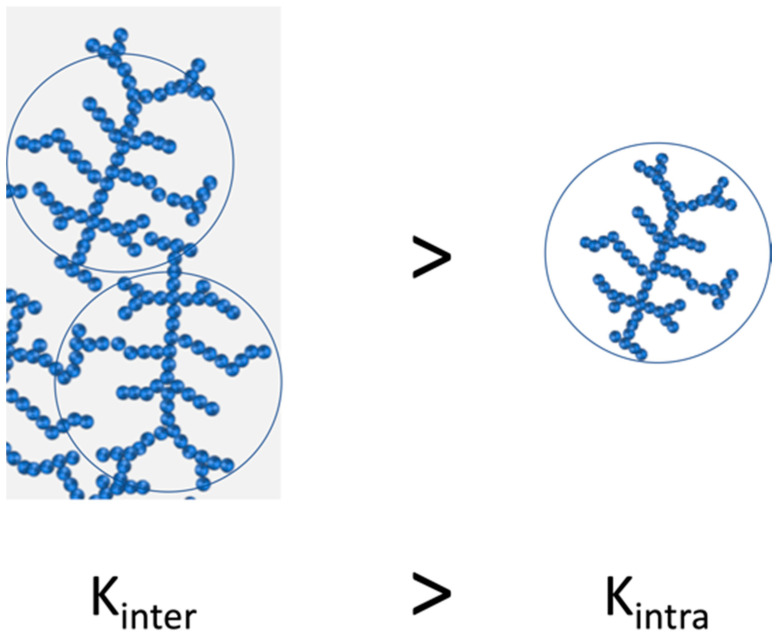
Representation of the strong-link regime, where the elastic constant between aggregates (inter-floc) is higher than the elastic constant between particles within the aggregate.

(a) Strong-link regime:

In this regime, it is considered that the interactions between aggregates (inter-floc) have a higher elastic constant than between the particles (intra-floc) (Scheme 2).

Thus, the macroscopic elastic constant *K* and, hence, elastic modulus plateau *G*′_0_ are driven by the elastic constant between aggregates *K_ξ_*. By combining Equations (1), (4) and (5), the following equation is obtained:

(6) $$K=G_{0}^{\prime }\sim \varphi^{\left(d+x\right)/\left(d-D\right)}$$

The physical concept of this equation indicates that the elastic constant of a colloidal gel, represented as the elastic modulus plateau *G*′_0_, increases with the concentration or volume fraction following a power-law behavior with an exponent of (*d* + *x*)/(*d* − *D*). In this strong-link regime, the interactions among clusters/agglomerates (inter-floc) are tougher compared to the interactions among particles within the colloidal particles (intra-floc). Therefore, when the system is sheared, Δ*L*, then the agglomerate deformation is described as follows:

(7) (ΔL)ξ=ΔLLξ

Considering the force within an aggregate as:

(8) Fξ=Kξ(ΔL)ξ=K0ξ.2+x ΔLLξ

and the breakage of a single joint at a certain force (p.e.: *F_ξ_* = 1), then it is possible to determine the critical deformation value corresponding to the yield strain, the limit of linearity of the linear viscoelastic range (LVR), γ_0_ as:

(9) $$\gamma_{0}=\frac{\Delta L}{L}\;\sim \;\xi^{1+x}\sim \varphi^{-\left(1+x\right)/\left(d-D\right)}$$

This equation predicts that the linear viscoelastic range diminishes with the increase in volume fraction.

**Scheme 3:**
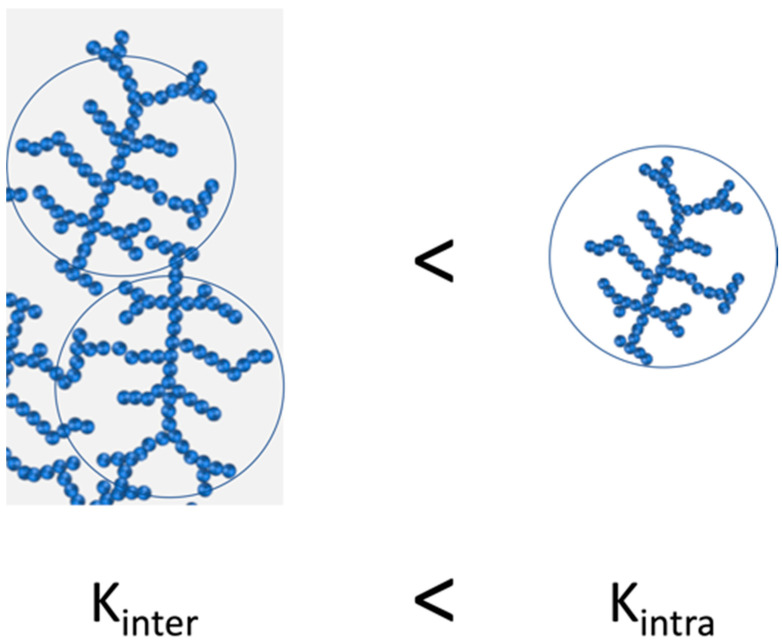
Representation of the weak-link regime, where the elastic constant within the aggregates (intra-floc) is higher than the elastic constant between aggregates.

(b) Weak-link regime:

In this regime, the interactions within the aggregates (intra-floc) are stronger than the bonds between the aggregates (inter-floc), indicating that the elasticity of the system is driven by the elasticity of the interactions between aggregates *Ka* (see Scheme 3).

When the elastic constant of an *L* size system is represented through the elastic constant of the aggregates (flocs), from the Equation (5) it is obtained that:

(10) $$K\sim {\left(\frac{L}{\xi }\right)}^{d-2}K_{a}\;\sim \;\xi^{-\left(d-2\right)}={G\prime }_{0}\sim \varphi^{\left(d-2\right)/\left(d-D\right)}$$

Similarly, the limit of viscoelastic regime, γ_0_, is also obtained

(11) $$\gamma_{0}\sim \;\xi^{-1}\sim \varphi^{1/\left(d-D\right)}$$

The comparison of both regimes evidenced that in the weak-link regime the elastic constant of the system decreases more slowly compared to the strong-link regime. Additionally, in the weak-link regime it is predicted that there is an increase in the linear viscoelastic range with the increase in particle volume fraction.

#### 3.2.2. Wu and Morbidelli Model

The model developed by Shih et al. describes two extreme situations; but the transition between both regimes can lead to intermediate behaviors in which both interactions between the aggregates (inter) and within the aggregates (intra) contribute to the overall elasticity of the colloidal gel. That is why Wu and Morbidelli [109], based on the development carried out by Shih et al., proposed a model in which a gradual transition from one model to another is also considered, giving rise to a more representative model.

For that, Wu and Morbidelli considered the aggregates as microscopic elements forming a macroscopic system. This distinction was made taking into account that the sizes of the aggregates forming the colloidal gel range from one to hundreds of microns. Therefore, based on this consideration, they divided the macroscopic system into two levels of structure: intra- and inter-microstructure that would correspond to the aggregates and the agglomerate (set of aggregates), respectively, resulting in the following expression that describes a constant of effective elasticity,

(12) $$\frac{1}{K_{eff}}=\frac{1}{K_{\xi }}+\frac{1}{K_{a}}$$

with *K_ξ_* and *K_a_* being the elastic constant of intra- and inter-microstructures, respectively.

Taking into account this division of structure levels, the macroscopic elastic constant *K*, Equation (5), is expressed in this way,

(13) $$K\propto {\left(\frac{L}{\xi }\right)}^{d-2}\frac{K_{\xi }}{1+\left(K_{\xi }/K_{a}\right)}$$

To be able to apply the above equation, the denominator needs to be converted into a scale law-type relationship,

(14) $$\frac{K_{\xi }}{1+\left(K_{\xi }/K_{a}\right)}≅{\left(\frac{K_{\xi }}{K_{a}}\right)}^{\alpha }$$

where α is a constant in the range of [0,1]. Therefore, substituting Equation (4) into Equation (13), the macroscopic elastic constant is expressed as follows,

(15) $$K\propto {\left(\frac{L}{\xi }\right)}^{d-2}\;K_{\xi }{\left(\frac{K_{\xi }}{K_{a}}\right)}^{\alpha }$$

**Scheme 4:**
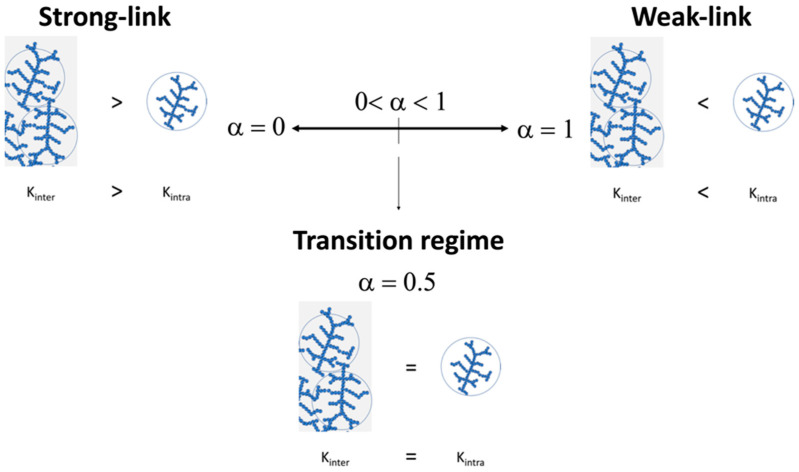
Representation of the physical meaning of α parameter where both the strong and weak-link regimes as well as the transition regime are considered.

If *K_a_* >> *K_ξ_*, then α = 0 and the macroscopic spring constant *K* would correspond to the strong-link regime determined by Shih et al. In contrast, when *K_a_* << *K_ξ_*_,_ then α = 1, which would correspond to a weak-link regime. Intermediate regimes are obtained for α values within a range of 0 < α < 1 (Scheme 4).

Wu and Morbidelli [109] used the development of Shih et al. [108] and the Kantor and Webman [107] relationship again to find the scale relationship between the elastic constant and the size of the aggregates. For that, it was considered that *Ka* is independent of *ξ*. Thus, the elastic constant is defined as:

(16) $$\;K_{\xi }\sim {\frac{1}{\xi^{2+x}}}^{\;}$$

By substituting Equation (16) into Equation (15), it is obtained that,

(17) $$\;K_{\xi }\sim {\frac{1}{\xi^{\beta }}}^{\;}$$

where

(18) β=(d−2)+(2+x)(1−α)

*β* is a constant that, for a three-dimensional system *d* = 3, has a value between 1 for α = 1 and 3 + *x* for α = 0.

When substituting Equation (1), which relates in a scalar fashion the elastic constant and the particle concentration, into Equation (17), the relationship between elastic constant (or elastic modulus plateau *G*′_0_) is related to the fractal dimension as follows:

(19) $$K=G_{0}^{\prime }\sim \varphi^{\beta /\left(d-D\right)}$$

The next step is to obtain the scale relationship between the critical strain limit (yield strain) and the fractal dimension. Assuming again that a macroscopic strain is applied in an *L* size system, the deformation corresponding to an aggregate is represented in Equation (8).

Considering the *K_eff_* defined in Equation (12) and applying Equations (5) and (14), the force exerted in an aggregate can be described as:

(20) Fξ=Kξ(ΔL)ξ=Kξ(KξKa)αΔLLξ∝1ξ(2+x)(1−α)−1 (ΔLL)

Assuming that a single link breaks at a certain force (p.e: *F_ξ_* = 1), then the limit of linearity, γ_0_, can be described as follows:

(21) $$\gamma_{0}=\frac{\Delta L}{L}\;\sim \;\xi^{\left(2+x\right)\left(1-\alpha \right)-1}\sim \varphi^{\left(d-\beta -1\right)/\left(d-D\right)}$$

Therefore, from these equations it is possible to determine from the experimental data the elastic constant *K* or *G*′_0_ and the critical deformation γ_0_ and to calculate both the fractal dimension and α parameter, which allow determining the type of link regime of the system.

**Figure 4:**
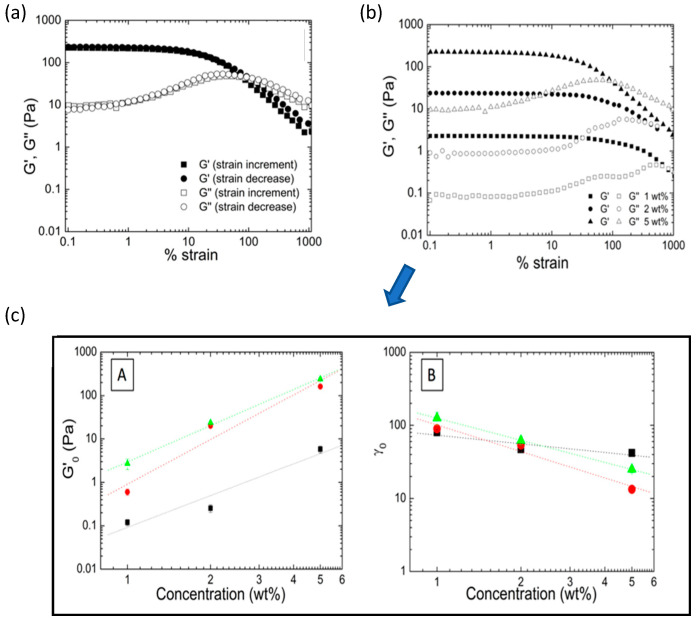
(**a**) Evolution of elastic (*G*′) and viscous (*G*″) modulus as a function of % of strain determined as the strain increases and decreases. (**b**) Strain sweep test corresponding to microgel aqueous dispersion (1, 2, and 5 wt%) containing 10% of AuNP. (**c**) Double-logarithmic plots of the elastic modulus plateau *G*′ (**A**) and critical strain γc (**B**) as a function of microgel concentration. Adapted from Echeverria et al. [115]; licensed under CC BY 4.0 (open access).

There are several studies regarding the interplay of microgel inter-particle interaction modifications by changing particle size, surface charge (functionalization), and cross-linking degree [110,111,112,113,114]. Most of them used poly(N-isopropylacrylamide) PNIPAM microgels. However, reports aimed to evaluate, control, and predict its influence on the fractal structure and cluster formation through rheology are scarce. For instance, we performed a detailed rheological study of hybrid poly(acrylamide-co-acrylic acid) aqueous microgel dispersion [6]. Our intention was focused on understanding how the presence of metallic nanoparticles (gold nanoparticles (AuNP)) [115] and metal oxide nanoparticles (superparamagnetic Fe_3_O_4_) [116], embedded within the microgel matrix could affect the viscoelastic properties and the colloidal gel structure formation, among other objectives. Both types of nanoparticles act as fillers, reinforcing the polymeric matrix and giving rise to higher values of elastic moduli (*G*′). By analyzing the behavior under deformation (strain sweep tests) of both hybrid microgel systems, it could be confirmed that the colloidal gel structure breaks upon deformation and completely restructures as the deformation gradually disappears (Figure 4a). In order to go deeper into such structure formation, both Shih et al. and Wu and Morbidelli scaling models were applied. Strain sweep tests at different microgel concentrations were performed (Figure 4b). The obtained elastic modulus plateau (*G*′_0_) and the yield strain (γ_0_) were represented in a double logarithmic plot vs. microgel concentration (Figure 4c), and the slopes were used to calculate the fractal dimensions and the type of interaction regime. It was concluded that the encapsulation of either AuNP (5 and 10 wt%) [115] (Figure 4) or Fe_3_O_4_ (5, 10, and 15 wt%) [116] contributed to increase the inter-floc interactions, thus hampering the formation of clusters or agglomerates. Accordingly, the estimated fractal dimensions, *D*, of both hybrid microgel dispersions decreased from 2.3–2.4 to 1.2 with the nanoparticles’ content from 5 to 10–15 wt%, respectively (Table 1). This remarkable decrease in the fractal dimension evidenced that the incorporation of either AuNP or Fe_3_O_4_ nanoparticles changed the growth/aggregation mechanism of the aqueous dispersions, from RLCA to DLCA, resulting in the formation of agglomerates with looser structures at rest that are easily deformable [115,116].

**Figure 5:**
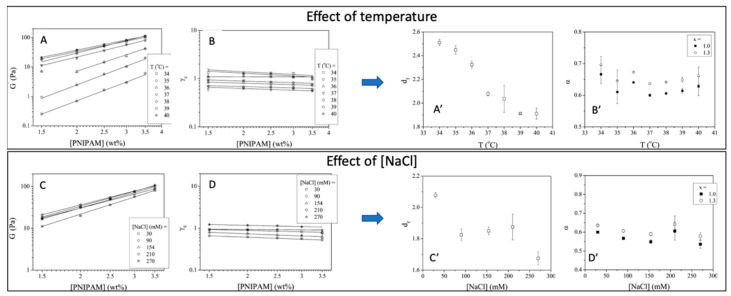
Graphs on the left: double-logarithmic plots of the elastic modulus *G*′ (**A**,**C**) and critical strain γc (**B**,**D**) of the hydrogels gelled at various temperatures and various [NaCl], as a function of microgel concentration. Graphs in the right: (**A’**,**C’**) fractal dimension, df, and (**B’**,**D’**)) the parameter, α, of the in situ-formed gels gelled at various temperatures calculated using the Wu and Morbidelli model. Adapted with permission from [118], copyright 2012 American Chemical Society.

Additional examples of the use of fractal analysis can be found in the literature. Guan et al. [117,118] developed hydrogels based on thermoresponsive PNIPAM microgels used as building blocks to construct an injectable thermal gelling scaffold for 3D cell culture. These hydrogels were formed in the presence of Ca^2+^ in order to induce changes in the inter-particle interaction. By applying both Shih et al.’s and Wu and Morbidelli’s rheology models, the fractal analysis of the hydrogels revealed that salt concentration as well as temperature affected the interactions among microgels (see Figure 5).

More recently, Echeverria et al. also attempted to modify PNIPAM-DMAEMA microgels’ colloidal gel behavior through quaternization reaction, although no relevant differences were obtained [119].

To have knowledge of the rheological behavior might serve as a tool to predict and control the effect of composition (cross-linker degree, cross-linker distribution, nanoparticles’ content, etc.). Varying the microstructure of the microgel, suspensions with different rheological properties can be obtained as described in the recent research by Dieuzy et al. [120]. In this work, the effects of different cross-linkers (oligo(ethylene glycol) diacrylate (OEGDA) and N,N-methylene bis-acrylamide (MBA)) on poly(oligoethylene glycol) methacrylates (POEGMA)-based thermo- and pH-responsive microgels on the rheological behavior were studied. In order to do so, they combined rheology tests and ^1^H NMR transverse relaxation measurements. Rheology tests (zero-shear viscosity and long time creep test) indicated that microgels behave more like hard spheres. Additionally, the effective volume fraction was considered as an appropriate scaling factor to compare microgels to the hard sphere behavior. The authors confirmed that viscosities of the studied microgel dispersions superimposed and reduced into a single curve following a hard sphere model. The ^1^H NMR transverse relaxation measurements confirmed the core-shell microstructure of microgels using both cross-linkers. But microgels prepared using MBA ended up in core-shell microgels with looser shells and less restricted dangling chains at the edge.

In a more recent work, Gosh et al. studied the mechanics of self-cross-linked, slightly charged, repulsive PNIPAM microgel suspensions over a very wide range of concentrations, going from the fluid, glassy, and “soft jammed” regimes [121]. From the combination of theoretical and linear/non-linear rheology studies, they were able to predict quiescent relaxation and yield strain under deformation as a function of microgel concentration. The final goal of the work was the use of this approach as a tool for the characterization of other microgel suspensions (based on different chemistries) in order to predict packing structures, shear elastic modulus, structure relaxation, and non-linear rheology, which are crucial for their further performance in the different potential applications.

The information and control over the composition and microgel microstructure and, therefore, over the rheological properties, are very relevant since they could facilitate a further self-assembly, for instance, toward scaffold formation applicable in tissue regeneration [122,123,124] or as biomaterials for 3D printing and cell culture applications [125]. An example of this can be found in the recent work of De Rutte et al. [123], in which they describe an approach to fabricate highly uniform bioactive microgel building blocks in a continuous scalable way, allowing the formation of highly modular microporous scaffolds for cell and tissue growth. In addition to this, to have control over the viscoelastic properties also facilitates the development of injectable systems. Microgels can assemble and promote injectability. Microgel suspensions can be injected and further assemble in situ in the required location [126,127]. This assembly can also occur through reversible dynamic bonds such as electrostatic interactions or hydrogen bonds, with a shear-thinning behavior so that microgels can flow, having a liquid-like behavior but above certain (injection) forces evolve toward a gel-like behavior [128].

### 3.3. Recent Advances on the Characterization of Microgels’ Single-Particle Interactions

**Figure 6:**
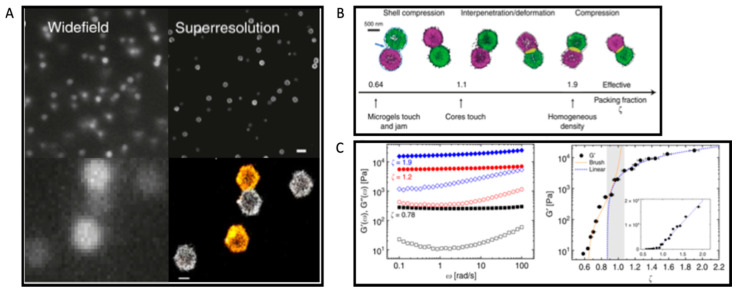
(**A**)**.** Images of PNIPAM microgels labeled with Alexa Fluor^®647^ dye, comparing conventional wide-field and dSTORM super-resolution images (two-color image) of glass sample (ζ = 1.89, c = 2.36 wt%) (scale bar, 2 µm in the upper images and 500 nm in the lower images) (**B**). Two-color microscopy of microgel pairs. Evolution of the interaction with the increase in the effective packing fraction ζ (from left to right, ζ = 0.86, 1.01, 1.26, 1.50, 1.89, 2.13). (**C**)**.** (left graph) Elastic modulus, *G*′(ω) (full symbols), and viscous modulus, *G*″(ω) (open symbols), as a function of frequency ω for different packing fractions, ranging from jammed to overpacked (ζ = 0.78 to 1.9). (right graph) Elastic modulus *G*′ at constant frequency (ω = 1.2 rad/s) as a function of packing fraction ζ fitted with the Brush model (orange, dotted line) at lower ζ and with a linear function at higher ζ (ζ ≥ ζc = 0.87). The shaded area marks the crossover between the regime controlled by shell compression to the one governed by core–core interactions. (Inset corresponds to the linear representation of the same data.) (Adapted from Conley et al.; licensed under CC BY 4.0 (open access) [139].

Many the of studies described in the review assumed microgels as spherical gel particles, and theories that are applied for polymer gels have been used to describe or predict elastic properties [3,103,121]. Nowadays, after years of research, the microgel particles are described as having a polymer gel core with a gradient of cross-linking, which decreases toward the outer side of the particle, and with dangling ends in contact with the solvent [129]. This particular morphology or microstructure is responsible for the ability to deform/compress microgels when interacting above a certain volume fraction (concentration). However, despite all the models, experimental work, and studies carried out on the rheological properties of microgels [130,131,132,133,134], there is still the need to build a widely accepted outline that could cover the entire range of packing densities, from glassy to jamming (or paste) up to highly compressed states (or overpacked states), and the interactions occurring among particles within each packing state. In order to go deeper in this aspect, and helped by the latest advanced characterization techniques, recent research has been focused on the study of microgel interactions at a single-particle nanoscale level based on zero average contact small-angle neutron scattering [135] and super-resolution microscopy [136,137,138]. A recent example of this can be found in the relevant work of Conley et al., where they studied the viscoelastic properties of a swollen, thermoresponsive PNIPAM-based microgel suspension having a dense core and a fuzzy shell, combined with the observation and analysis of the individual particle structure and morphology through super-resolution microscopy [139]. The objective of the research was to connect the mechanism beyond the viscoelastic properties of a microgel suspension, with the interactions occurring within microgels in the different concentration regimes (glassy, jamming, and compressed regimes). The structure and morphology of PNIPAM microgels and a pair of microgels were observed by means of super-resolution microscopy (Figure 6A) detecting three consecutive stages of packing, from jammed to overpacked (see Figure 6B) [137]. In the first stage, the shell of the microgel started to be compressed. However, as soon as the cores of the microgels made contact, microgel shell inter-penetration occurred and the microgels started to deform. This allowed a denser packing of microgels. Such inter-penetration/deformation continued and increased as the contact surface expanded. In the last stage, the remaining interaction mechanism within the microgel pair is the compression, resulting in the reduction in size of the swollen microgel. To connect these results with viscoelastic properties, frequency sweep tests were performed at different volume fractions (from jamming to overpacked). As shown in Figure 6C (left graph), all suspensions described an elastic modulus *G*′ almost independent of the frequency and being greater than *G*″, which is indicative of a solid-like behavior. To better understand the effect of volume fraction, the authors collected the *G*′ values at a constant frequency and represented as a function of the volume fraction (Figure 6C, right graph). Two differentiated behaviors marked by the shaded area were observed. Below the shaded area, which is the jamming packing fraction, the measured elastic modulus is weak, and it was associated with an entropic glass regime. Beyond this area, the elasticity was driven by the jamming elasticity, showing an increase in *G*′ of almost 3 orders of magnitude. However, as the volume fraction increased toward the overpacked state, the trend tended to stabilize and the slope reduced substantially. Definitively, among the major conclusions drawn from this research is that the particulate nature of microgel suspensions dominates the behavior also at their highest packing fractions. Additionally, microgels shared some properties with emulsions but entered into different regimes due to the deformation/compression ability up to the overpacked regime.

## 4. Conclusions

Microgels have significantly evolved and contributed to the development of advanced materials. Such evolution has arisen from the control over the microgel synthesis, which allowed the design of different microgel architectures: core-shell, multicore-shell, and hollow microgels, in addition to the development of controlled (multi-) stimuli-responsive microgels. Undoubtedly, microgels owe their properties mainly to their complex morphology (and architectures), inter- and intra-particle interactions, and further packaging, which give rise to a wide variety of new physical states and behaviors.

This brief review presented a comprehensive overview about the rheology and applications of stimuli-responsive microgels; it evidences the importance of rheological characterization and the application of scaling theories, such the Shih et al. and Wu and Morbidelli models, which allow us to indirectly determine the type of interactions occurring among stimuli-responsive (temperature, pH) microgel particles within a dispersion. The rheological behavior has been used in some examples of stimuli-responsive microgels and hybrid microgels to predict elastic properties, quiescent relaxation, and yield strain. These fractal models are appropriate for determining how changes in superficial charge, external stimuli, and microgel composition as well as inorganics’ particle incorporation might affect the interactions among microgel aggregates at different concentrations. However, they do not provide information directly related to single microgel particle interactions; this is of importance for further microgel applications as drug delivery systems, building blocks that can self-assemble to form structures/scaffolds for tissue regeneration, and also as biolubricants, due to the physical nature of these kinds of polymeric systems. Microgels’ deformability and compressibility (viscoelastic properties) allow us to reach non-spherical shapes so that they can densely pack to form 3D structures (fractals) and scaffolds or undergo inter-penetration of their external shell (in a core-shell microgel architecture). In this regard, we have also described the most recent works done on rheological characterization of microgel single-particles’ interactions combining rheology with advanced technologies such as super-resolution microscopy.

Above all, this brief review showed that, although microgels have been researched for a long time, there is still room for knowledge, particularly from the point of view of the rheology that has been proven to be crucial for the development and correct performance of different applications such as the ones described in this review. In line with this, we believe that the future lines of rheological characterization of microgels will lie in filling the gap between the characterization of microgels at the colloidal level and the characterization at the single-particle level. This knowledge will allow the development of new biomedical applications, especially injectable systems and also safer materials. For instance, being able to tailor how stimuli-responsive microgels should behave under deformation, and at rest, would avoid undesirable or unexpected agglomeration problems. These could limit their responsiveness or have dramatic consequences such as blood clots when referring to real, in vivo use of the smart dispersions.

## Figures and Tables

**Table 1:** Summary of the results obtained by applying Shih et al. and Wu and Morbidelli models to microgels’ systems containing Au and Fe_3_O_4_ nanoparticles [115,116].

Samples	Slopes		Shih et al.		Wu and Morbidelli		Regime
	A	B	D	x	D	α	
MG	2.56	−0.22	---	<0	2.14	0.63	Transition (weak)
MG-5%AuNP	3.82	−0.62	---	<0	2.3	0.57	Transition (weak)
MG-10%AuNP	2.29	−1.18	1.19	1.13	1.2	0.04	Strong-link
MG-5%Fe3O4	2.27	−0.82	n.a	n.a	1.62	0.35	Transition (strong)
MG-10%Fe_3_O_4_	2.04	−0.50	n.a	n.a	1.70	0.50	Transition
MG-15%Fe_3_O_4_	1.43	−0.29	n.a	n.a	1.24	0.53	Transition
